# Peer Review of “Cognitive Factors Associated With Public Acceptance of COVID-19 Nonpharmaceutical Prevention Measures: Cross-sectional Study”

**DOI:** 10.2196/37378

**Published:** 2022-05-13

**Authors:** Sina Azadnajafabad

**Affiliations:** 1 Non-Communicable Diseases Research Center Endocrinology and Metabolism Research Institute Tehran University of Medical Sciences Tehran Iran

**Keywords:** Extended Parallel Process Model, COVID-19, lockdown, public acceptance, nonpharmaceutical measures, Likert scale, France

*This is a peer-review report submitted for the paper “Cognitive Factors Associated With Public Acceptance of COVID-19 Nonpharmaceutical Prevention Measures: Cross-sectional Study.”*

## Round 1 Review

### General Comments

This paper [1] tried to explore the acceptance of the COVID-19 preventive measures and their relationship with perception of the disease via an online survey among the French population. The overall drafted manuscript is acceptable; however, the notion of the paper is a little outdated and numerous publications regarding the COVID-19 are available now that used this method. Evaluation of policies like mask use mandates is not a hot topic now and may not add much to the evidence.
